# New data on the distribution and diversity of the Tonkin limestone rat (*Tonkinomysdaovantieni*, Rodentia, Muridae)

**DOI:** 10.3897/BDJ.11.e110335

**Published:** 2023-09-27

**Authors:** Alexander E Balakirev, Phuong Xuan Bui, Viatcheslav V Rozhnov

**Affiliations:** 1 A.N. Severtsov Institute of Ecology and Evolution, Russian Academy of Sciences, Moscow, Russia A.N. Severtsov Institute of Ecology and Evolution, Russian Academy of Sciences Moscow Russia; 2 Joint Russian-Vietnamese Tropical Research and Technological Centre, Hanoi, Vietnam Joint Russian-Vietnamese Tropical Research and Technological Centre Hanoi Vietnam

**Keywords:** Southeast Asia, Vietnam, rodents, taxonomy, biodiversity

## Abstract

The paper presented novel findings of little-known species of rodents the Tonkin limestone rat *Tonkinomysdaovantieni* in Cao Bang Province, Vietnam with its morphological and genetic characterisation. The study summarises data on the distribution of this data-deficient species, available museum collections, genetic samples, information on its taxonomy and ecology, important to establish the proper conservation status of the species. An exhaustive map of the findings is provided. It is shown that, based on the data currently available, the species does not require taxonomic revision and also, apparently, does not need a special conservation measure; its status may be established to date as Near Threatened B1a+2a and the current population trend – Stable, IUCN.

## Introduction

The genus *Tonkinomys* Musser, Lunde & Son, 2006 was first described by Guy Musser (Musser et al. 2006) about 17 years ago from Lang Son Province, north-eastern Vietnam. The genus belongs to the muroid rodents (Rodentia, Muridae), subfamily Murinae. The holotype is currently held in the American Museum of Natural History (AMNH: 275618). As can be estimated today a posteriori, the first two specimens of this species (both females) were obtained even earlier from the same type locality by J.L. Eger, B.K. Lim and L. Mitchell as early as 2 May 2000; they were genotyped and listed in the BOLD system (Barcode of Life Data Systems, http://www.boldsystems.org, Hebert et al. (2003), Ratnasingham and Hebert (2007)) under BOLD identification numbers BOLD:AAH3527 and BOLD:AAH3528. However, these samples and sequences of the COI gene derived from them were taxonomically misinterpreted in the absence of comparative material and still treated in the GenBank databases as belonging to *Rattus*. Today, their close identity (99.84%) with the samples obtained from the same point (Balakirev et al. 2012, Balakirev et al. 2013) does not allow us to doubt their belonging to the same species.

The genus is currently considered as monotypic and available materials and data on distribution of the only known species *T.daovantieni* Musser, Lunde & Son, 2006 are mainly limited to the type locality (Wilson et al. 2017). The authors (Balakirev et al. 2012, Balakirev et al. 2013) first described the karyotype of the species and, based on mitochondrial (Cyt *b*, COI) and nuclear (IRBP) genes, initially investigated evolutionary relationships within the *Dacnomys* division and, in particular, between Tonkin limestone rats (*Tonkinomys*) and Laotian limestone rats (*Saxatilomys*), which made it possible to demonstrate the close similarity between these groups. Both *S.paulinae* and *T.daovantieni* have very similar ecological preferences, which, apparently, reflect their close phylogenetic relationship. Both species inhabit moist evergreen broadleaf forested habitats subsisting over limestone karst formations that are widespread in Laos and northern Vietnam (Musser et al. 2006, Wilson et al. 2017, Marsh et al. 2022). A few years ago, the species was also found in China (Cheng et al. 2018), exactly, in Málìpō County, Wenshan Prefecture, Yunnan Province, from where four additional specimens were obtained, caught near the Jīnсhǎng, Bābù and Mǎjiē villages. Unfortunately, this paper was published by the colleagues in Chinese without translation and, therefore, is still little known to a wide range of researchers. The distribution (Marsh et al. 2022) and conservation status (Laginha Pinto Correia 2016) of the species have not been updated since 2016 and still record the only type locality. Therefore, our purpose was to present new actual information about the natural range and intraspecific diversity of this rare (Laginha Pinto Correia 2016) and poorly-studied mammalian species.

## Material and methods

**Figure 1.**
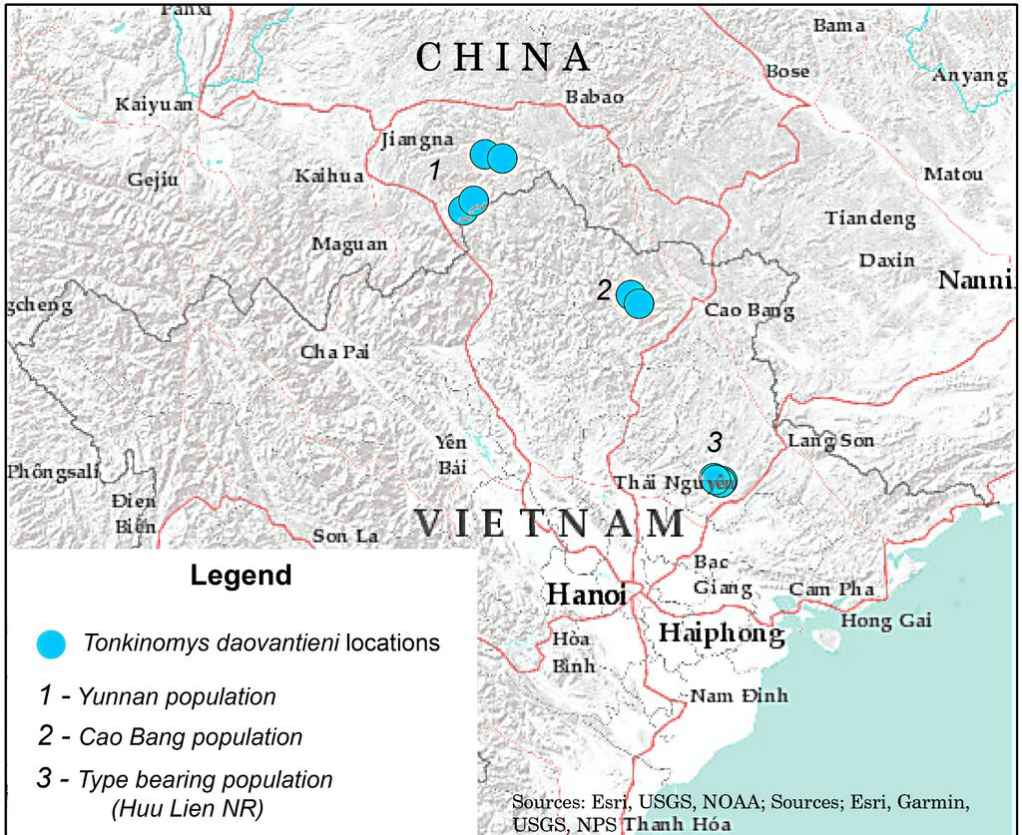
Currently documented *Tonkinomysdaovantieni* distribution map. Coordinates for Yunnan population are corrected in agreement with actual positions as referenced in Google Map for corresponding verbatim localities.

The animals were trapped by snap traps during one of the recent theriological expeditions organised by Russian-Vietnamese Tropical Research and Technological Center in Cao Bang Province, Nguyen Binh District, within Ca Thanh, Quang Thanh and Huong Dao communes, about 120 km apart to north-northeast from terra typica of this species (Huu Lien Nature Reserve, Lang Son Province, Vietnam). The field works were carried out in the period 3-18 February 2023. In total, three individuals of *T.daovantieni* (one adult male, one adult female and one young adult male) were obtained in Ca Thanh Commune (22.704907N, 105.835610E) and near Tinh Tuc Town (22.656775N, 105.883298E). They were used to obtain both morphological (skulls and skins) and genetic samples, (Fig. 1). The whole bodies were preserved in 70% ethanol and the skulls and skins of these animals are deposited now in the Zoological Museum of Moscow State University, Moscow (ZMMU, S-209166, S-209167 and S-209183).

The following twenty-three external measurements have been done for samples in field and in laboratory after skulls extraction and preparation by boiling in water for 5 min. Final skulls treatment have been made by bugs (*Dermestes* sp.) from the ZMMU collection and cranial characters were taken for each skull using digital calipers to the nearest 0.01 mm:

Head and body length (LHB), tail length (LT), hind foot length (LHF), length of ear loop (LE), body weight (BW), occipitonasal length (ONL), zygomatic breadth (ZB), interorbital breadth (IB), length of rostrum (LR), breadth of rostrum (BR), breadth of braincase (BBC), height of braincase (HBC), breadth of zygomatic plate (BZP), length of diastema (LD), length of incisive foramina (LIF), breadth of incisive foramina (BIF), length of bony palate (LBP) (palatal bridge), breadth across bony palate at first molars (BBP), postpalatal length (PPL), breadth of mesopterygoid fossa (BMF), length of bulla (LB), crown length of maxillary molar row (CLM^1-3^), breadth of first upper molar (BM^1^) following Musser and Newcomb (1983) and Musser et al. (2006).

As can be summarised for all available data, 37 individual samples and 15 genotyped ones are currently listed in international databases for three museums collections (Table 1).

Small pieces of liver were stored in 96% molecular grade ethanol and used for the DNA extraction. The total genomic DNA was extracted from piece of liver or muscules using a routine phenol/chloroform/proteinase K protocol (Kocher et al. 1989, Sambrook et al. 1989). All three new individuals have been genotyped by the full long Cyt *b* and growth hormone receptor partial sequence gene (GHR, 820 bp) and analysed together with six individuals obtained earlier (Balakirev et al. 2013) from terra typica (KC209558–KC209569). Universal routine PCR protocols have been used to amplify mtDNA fragment as follows: initial denaturation for 1 min 30 sec at 95°C, denaturation for 30 sec at 95°C, annealing for 1 min at 52°C and elongation for 45 sec at 72°C, followed by terminal elongation for 3 min at 72°C. The PCR reaction was performed in a 25µl volume that contained 2.5-3 mkl 10 x standard PCR buffer (Fermentas), 50 mM of each dNTP, 2 mM MgCl_2_, 10 pmole of each primer, 1 unit of Taq DNA polymerase (Fermentas) 20-50 ng of total DNA template per tube. The *Cyt b* gene was amplified directly using primers pair L14724A (Irwin et al. 1991, 5’-CGAAGCTTGATATGAAAAACCTCGTTG-3' and H15915, 5’-GGAATTCATCTCTCCGGTTTACAAGAC-3' (Kocher et al. 1989). For the COI gene, we sequenced the one time routine BOLD primers LCO1490 and HCO2198 (5'-GGTCAACAAATCATAAAGATATTGG-3' and 5'-TAAACTTCAGGGTGACCAAAAAATCA-3') and protocols have been used as explained in Hebert et al. (2003). The GHR gene was amplified by the two-round nested scheme explained in Jansa et al. (2009) with primers GHRF1, 5’-GGRAARTTRGAGGAGGRGAACACMATCTT; GHRF50 5’-TTCTAYARYGATGACTCYTGGGT-3'; GHR930R, 5’-RTAGCCACANGANGAGAGRAA-3'; and GHRendAlt 5’-GATTTTGTTCAGTTGGTCTGTGCTCAC. The double-stranded DNA products were directly sequenced in both directions using an ABI PRISM 3730xl Genetic Analyzer (Applied Biosystems, USA) and the BigDye® Terminator v.3.1 Cycle Sequencing Kit (Life Technologies Corporation, Carlsbad, CA,USA) in agreement with the manufacturer’s protocol.

We also analysed four Cyt *b* sequences (MG865970-MG865973) from Yunnan population (Cheng et al. 2018) and two more sequences (MG189667, MG189683) for another specimen from Huu Lien (Fabre et al. 2017). Therefore, this dataset combines all bulk of genetic information currently available for this species for all populations currently discovered. A number of related taxa belonging to the Dacnomys division, namely *Niviventer* (KY068809, KY068810, EF053025, EF053026), *Chiromyscus* (KF154039, KF154040), *Leopoldamys* (JX573331, JX573334) and *Saxatilomys* (JQ755867-JQ755867) were used as outgroups (Table 2).

Sequencing analyses, based on complete cytochrome *b* gene sequences, were conducted in MEGA X (Kumar et al. 2018). The evolutionary history was inferred by the Maximum Likelihood method and General Time Reversible model (Nei and Kumar 2000) as they are the most complex, universal and need no initial presumption about the codon evolution mode. Analysis involved 24 nucleotide sequences including external groups of *Dacnomys* division members. The tree with the highest log likelihood (-2514.14) was used. The initial tree for the heuristic search was obtained automatically by applying the Neighbour-Joining and BioNJ algorithms to a matrix of pairwise distances estimated using the Maximum Composite Likelihood (MCL) approach and then selecting the topology with superior log likelihood value. A discrete Gamma distribution was used to model evolutionary rate differences amongst sites (5 categories +G, parameter = 1.8187). The rate variation model allowed for some sites to be evolutionarily invariable ([+I], 58.68% sites). All positions with less than 95% site coverage were eliminated, i.e. fewer than 5% alignment gaps, missing data and ambiguous bases were allowed at any position (partial deletion option) resulting in a total of 739 positions in the final dataset. Bootstrap values were calculated with 10000 iterations.

Evolutionary analyses were conducted in MEGA X (Kumar et al. 2018). Estimates of evolutionary divergence between *Tonkinomysdaovantieni* geographical populations were made by Nei and Kumar (Nei and Kumar 2000). The number of base substitutions (1140 positions) per site from averaging over all sequence pairs between groups are shown in Table 3. Standard error estimate(s) are shown above the diagonal. Analyses were conducted using the Maximum Composite Likelihood model (Tamura et al. 2004). The rate variation amongst sites was modelled with a gamma distribution (shape parameter = 1.8187).

## Data resources

Preserved bodies and prepared skulls and skins of animals are deposited in Zoological Museum of Moscow State University, Moscow (ZMMU, S-209166, S-209167 and S-209183). Genatic data for new samples of study are deposited to GenBank under IDs OR058873-OR058875 and OR136871-OR136878.

## Results

**Figure 2.**
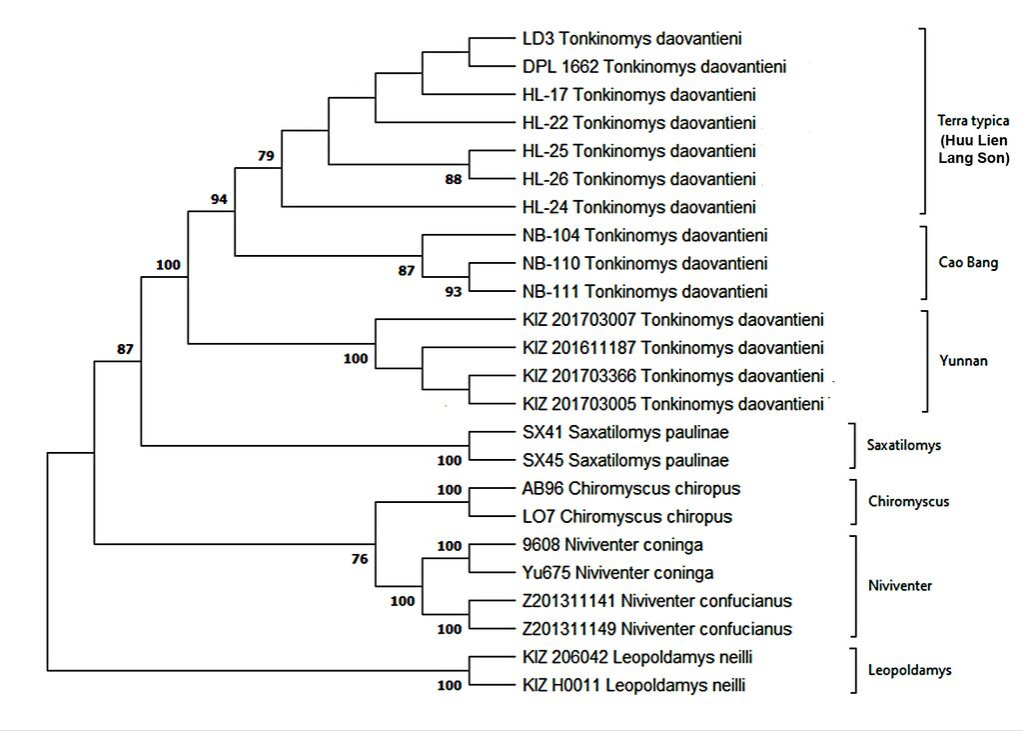
Phylogenetic tree of *Tonkinomysdaovantieni* constructed, based on Cyt *b* gene sequence. Footnotes: Bootstrap values are shown next to the branches (values lower than 70 not presented).

Molecular genetic analysis, based on the mitochondrial cytochrome *b* gene, showed that the new specimens form a monophyletic species clade with the samples obtained earlier. At the same time, new samples are reliably clustered as a sister group to the population from the type locality, but not with equally-distanced Yunnan ones (Fig. 2). The average genetic distance (*d*, K3P) between them is estimated as low as only about 0.9% which is characteristic for intraspecific divergence. At the same time, the genetic distance from the Yunnan population is appreciably higher, about 2.2-2.5%, but also cannot be treated as interspecific (Table 3). The bootstrap levels for the nodes of the tree between population clades constructed by the ML method are reliable. However, it can be seen that individuals from the new locality are actually from a single population with animals originating from the type locality. It can also be seen that this population exhibits higher haplotype diversity, which may indicate its pan-mixing. The genetic exchange is even better demonstrated by analysis of the nuclear gene for the growth hormone receptor gene (GHR). Thus, amongst four individuals genotyped for this gene, at least one heterozygote (sample NB-111 has ambiguous substitutes in 59 and 122 nucleotide positions as referenced in the fragment of the study) was found, which combines alleles of the gene characteristic of populations from terra typica and Cao Bang Province, indicating a genetic exchange between Vietnamese populations.

**Morphological peculiarities**

**External description**

**Figure 3.**
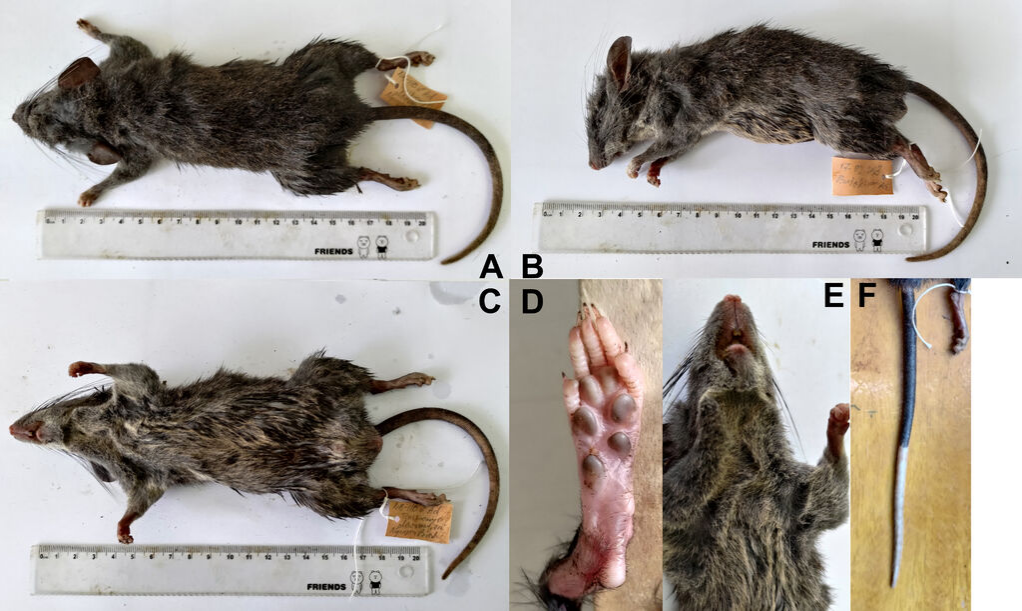
General appearance of *Tonkinomysdaovantieni*. Sample NB-110, adult male, museum voucher ZMMU S-209166, Tinh Tuc Town, Nguyen Binh District, Cao Bang Province, Vietnam. **A** Lateral view. **B** Side view. **C** Ventral view. **D** Plantar view of left foot. **E** Breast and throat. **F** Tail colouration pattern.

Below, we provide a brief description of the morphological features for the newly-found population of the Tonkin limestone rats, expanding on the previously-compiled original description of the species. This is a dark ash-coloured medium-sized rat, weighing up to 170 g (Fig. 3). The head and eyes are large, vibrissa area is fleshy, ears large, grey, slightly pubescent. Thick and shiny fur coat with guard hairs and dark underfur, slightly differing in colour on the dorsal and ventral sides of the body; however, the border of the colour transition is indistinct. The back is generally grey-black, the belly is lighter, ash-grey. The female has four pairs of nipples, one pair of thoracic, one pair of axillary and two pairs of inguinal. The feet and hands of the forelegs are thin, covered with dark grey short hairs; there are five plantar pads on the hands and six on the hind feet, their structure being similar to that described in the original description (Fig. 3D). The tail is relatively thick, with annular scales, slightly shorter than the length of the body, generally indistinctly two-coloured, dorsal side black-brown, ventral side of the tail light. The distal half or third of the tail is completely white, the border of the colour transition is clear (Fig. 3, Table 4) The tail is evenly covered with short hairs, the terminal brush is absent. Most individuals from terra typica (Huu Lien Nature Reserve, Lang Son Province, Vietnam) have more or less large white spots on the chest and forehead. However, individuals obtained from Cao Bang Province, as well as the populations from Yunnan (Fig. 3), do not have any spots (E). In addition, in the population from Cao Bang, individuals show a remarkable longer area of tail tip discolouration. Whereas for animals from typical and Yunnan populations, the white end of the tail occupies only 1/3, while the animals from Cao Bang exhibit a decolouredtip up to 1/2 of the tail length. It should also be noted that our animals are somewhat smaller than the typical population. In adults, the LHB is 169-190, LT 164-168, LHF 36-38 and LE 28-29 mm, all of which are noticeably smaller than indicated for the holotype, for which these values are 213, 177, 40 and 30 mm, respectively. As for their size characteristics, they correspond to the Yunnan population, whose sizes fall within the same limits.

**Skull morphology**

**Figure 4.**
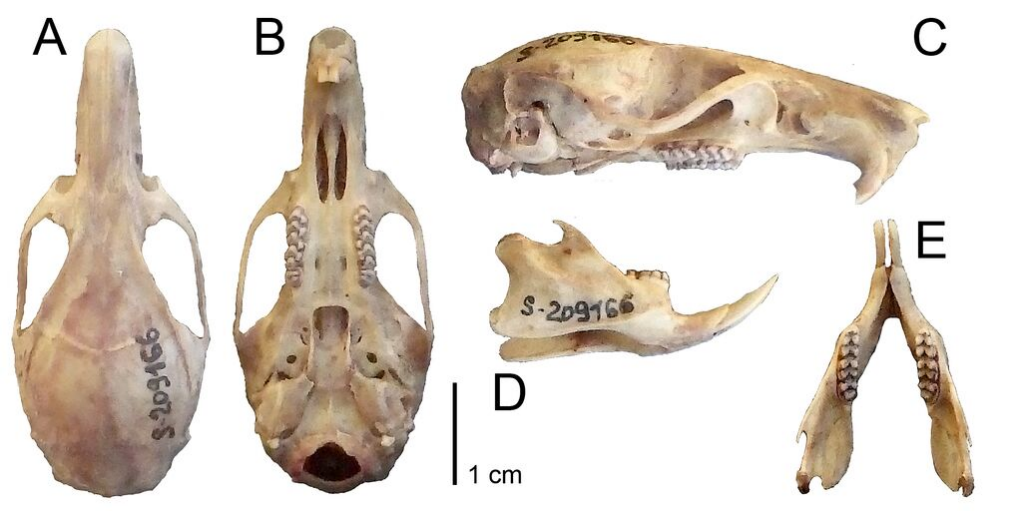
Skull of *Tonkinomysdaovantieni*. Sample NB-110, adult male, museum voucher ZMMU S-209166, Tinh Tuc, Nguyen Binh District, Cao Bang Province, Vietnam. **A.** Dorsal view. **B.** Ventral view. **C.** Side view. **D.** Lower jaw, side view. **E.** Lower jaw, dorsal view.

The skull of our specimens (Fig. 4) is generally of the typical shape for the species, with a narrow, long and pointed rostrum, with strong and wide zygomatic arches, fine and slender bony crests and a broad masseter plate. Incisal foramina are elongated, wide and rounded. Bulla compact, not swollen, lying freely, there are wide fissures between them and the pterygoid bones. The dental formula is typical for Muridae: 1.0.0.3/1.0.0.3 = 16 . The upper and lower incisors have a well-defined layer of light-yellow enamel on the frontside, the surface of the teeth is smooth, the incisors of the upper jaw are generally orthodontic. The molars have well-developed crowns. The chewing surfaces of molars consist of two parallel "V"-shaped ridges and isolated tubercles. The first upper molar has four roots, the second and third upper molars have three roots, whereas all lower molars have two roots. The basic measurements of the skull are summarised in Table 4.

## Discussion

**General remarks on ecology, taxonomy and conservation status of the species**

At present, *Tonkinomysdaovantieni* has been found within a rather extended area in northern-east Indochina in Vietnam and China stretching for at least 250 km from northwest to southeast where it is reliably recorded from at least ten individual localities representing three geographical populations. All of them are associated with ancient Devonian and Cambrian limestone karsts, at least 280 million years old, covered with forest vegetation. Based on our data on this species' captures and information from local people's interrogations, who occasionally use this species as an object of hunting, allows us to assert that, even within the range, in fundamentally suitable forest habitats, in places where there are no rocky outcrops found on the surface (karsts are buried deep under the surface soil), the species is not recorded. This indirectly indicates the ecological association of this species with limestone outcrops. The nature of such relationships cannot yet be considered reliably established; however, it can be assumed that this may be due to species ecological preferences in the choice of shelters and nesting burrows confined to numerous narrow karst cavities characteristic of ancient karsts. The Vietnamese populations are genetically close to each other and, apparently, pan-mictic, while the Yunnanese one represents a separate genetically unique sister group in relation to them, but apparently indistinguishable morphologically. The genetic distance from the Yunnan population is about 2.2-2.5% and it cannot be treated as interspecific. The level of genetic divergence in mitochondrial genes, as well as the general species polymorphism in the nuclear gene of study, does not allow us yet to distinguish any obvious taxonomic categories of species or subspecies rank within *Tonkinomys*. As for abundance of the species in nature and its conservation status, the information obtained to date indicates that the populations are sporadically distributed and genetically quite diverse. In habitats suitable for the species, they are not inferior or only slightly inferior in number to *Leopoldamys* and some species of *Niviventer* and *Chiromyscus* which are usually present in the same area.

In agreement with IUCN rules, there are five quantitative criteria that are used to determine whether a taxon is threatened or not and, if threatened, to which category of threat it belongs (Critically Endangered, Endangered or Vulnerable). These five criteria are:

A. Population size reduction (past, present and/or projected);

B. Geographic range size and fragmentation, few locations, decline or fluctuations;

C. Small and declining population size and fragmentation, fluctuations or few subpopulations;

D. Very small population or very restricted distribution;

E. Quantitative analysis of extinction risk (e.g. Population Viability Analysis).

Of the five circumstances given, only B can be applied to this species (Geographic range size and fragmentation, few sites, decline or fluctuations). The data currently available do not suggest either a very small population or any decline or reduction in population. To qualify for criterion B, the general distributional threshold must first be met for one of the categories of threat, either in terms of extent of occurrence (EOO) or area of occupancy (AOO). The taxon must then meet at least TWO of the three options listed for criterion B. The options are: (a) severely fragmented or known to exist in no more than “X” locations, (b) continuing decline or (c) extreme fluctuation (IUCN 2001, IUCN 2012). Of these criteria, only B1a is obviously applicable - fragmentation of the area due to the natural fragmentation of landscapes. Within category B, the species can formally be classified as VU (Vulnerable) according to criterion B1 (Extent of Occurrence EOO) and, according to subcategory B2a (Area of Occupancy AOO), it is possible even to be Endangered due to only three known populations and about ten localities currently identified. The range area of the species, estimated from an ellipse containing all known finds, covers about 18,500 km^2^. At the same time, on the one hand, the real area of karst formations within this zone is 4-5 times smaller, on the other hand, there is every reason to believe that the species is distributed much more widely, since karst regions cover many hundreds of square kilometres in the region of northern Indochina and southern China. It should also be noted that, in accordance with the IUCN rules (Anonymous 2022) in the absence of any plausible threat for the taxon, the term "location" cannot be used and the subcriteria that refer to the number of locations will not be met. The species apparently shows neither a noticeable decrease in abundance (b) nor its significant fluctuations (c) in relation to the size of the range or the number of individuals.

There is subjectivity in the establishment of boundaries amongst the categories of risk (Collen et al. 2016). The category Near Threatened is applied to taxa that do not qualify as threatened now, but may be close to qualifying as threatened and to taxa that do not currently meet the criteria for a threatened category, but are likely to do so if ongoing conservation actions abate or cease. According to the rules of the IUCN Red List Categories (Anonymous 2022), this means that there is adequate data for the species to assess the degree of risk. A taxon is Near Threatened when it has been evaluated against the criteria, but does not qualify for Critically Endangered, Endangered or Vulnerable now, but is close to qualifying for or is likely to qualify for a threatened category in the future. We tend to believe that this is exactly the situation with respect to *Tonkinomysdaovantieni*, as the species is not directly threatened and does not show trends in the reduction of its range and abundance; however, the natural fragmentation of its range carries certain risks.

The conservation status of a species also depends on the presence or absence of specially protected natural areas within its range. With regard to *Tonkinomysdaovantieni*, in the territory of north-eastern Vietnam, at least eight protected areas are located within the species range or in the closest vicinity, namely Tay Con Linh, Du Gia, Bac Dai Son (Hà Giang Province), Bac Me, Phia Oac - Phia Den (Cao Bằng Province), Kim Hy, Ba Be (Bắc Kan Province) and Hưu Lien (Lang Son Province). For at least four more territories, namely Na Hang (Tuyên Quang Province), Than Sa (Thai Nguyen Province), Tam Dao (Vinh Phuc Province) and Ky Thuong (Quang Ninh Province), the presence of the species can be assumed, based on distribution of suitable type of habitats. In China, in the Yunnan and Guangxi Provinces, there are also a number of national reserves where the species is likely to be found sooner or later. These are first of all: Shiwandashan National Nature Reserve (NNR), Cenwanglaoshan NNR, Chongzuo White-headed Black Langur NNR (Guanxi), Nan'gunhe NNR, Xishuangbanna NNR, Ailaoshan NNR, Wuliangshan National Nature Reserve, Jinping Watershed NNR, Daweishan NNR, Wenshan NNR, Yaoshan NNR (Yunnan) and probably others. This circumstances are in favour of the protective status of this species and lower risks.

## Conclusions

Thus, we believe that the category Near Threatened B1a+2a and the current population trend – Stable are justified for this species.. The main threats to its conservation are not primarily linked to direct impacts and population reduction, but rather to its association with specific and highly-specialised habitat types, such as ancient karsts covered with forest vegetation, which may limit its potential distribution.

## Figures and Tables

**Table 1.** Geographical locations for *Tonkinomysdaovantieni* samples available (only geolocated samples listed). Geographical coordinates from Cheng et al. (2018) presented as indicated in the paper in spite of the fact that for two points (Ba Bu xiang, 23.223806N, 104.895069E and Ma Jie xiang, 23.456965N, 105.065682E), the coordinates are obviously wrong due to its falling in Vietnamese territory instead of China.

Voucher	N (dd.dddd)	E (dd.dddd)	H (m a.s.l.)	locality	Reference
NB-104	22.704907	105.835610	1200	Ca Thanh	this paper
NB-110	22.656775	105.883298	820	Tinh Tuc	
NB-111	22.656775	105.883298	820	Tinh Tuc	
LD-3	21.690819444	106.331305556	300-400	Lan Ðat village	Balakirev et al. (2013)
HL-17	21.666666667	106.372866667	300-400	Huu Lien NR	
HL-22	21.674722222	106.380833333	300-400	Huu Lien NR	
HL-24	21.666666667	106.372866667	300-400	Huu Lien NR	
HL-25	21.666666667	106.372866667	300-400	Huu Lien NR	
HL-26	21.666666667	106.372866667	300-400	Huu Lien NR	
KIZ 201703366	23.478889	104.962778	1 340	Majiexiang*	Cheng et al. (2018)
KIZ 201703007	23.163611	104.940556	1 290	Babuxiang*	
KIZ 201703005	23.164167	104.994167	1 290	Babuxiang*	
KIZ 201611187	23.170833	104.832778	1 525	Jīnchǎng*	
DPL 1662	21.680833	106.34111	150-400	Huu Lien NR	Fabre et al. (2017)
ROM:F48185	21.683	106.333	500	Huu Lien NR	J.L.Eger et al. (2000) unpublished

**Table 2.** *Tonkinomysdaovantieni* samples currently available and used for genetic analyses (marked in bold).

		GenBank ID		
Species	Voucher	*Cyt b*	GHR	COI
*Tonkinomysdaovantieni*	NB-104	OR058873	OR136871	OR058876
*Tonkinomysdaovantieni*	NB-110	OR058874	OR136872	OR058877
*Tonkinomysdaovantieni*	NB-111	OR058875	OR136873	OR058878
*Tonkinomysdaovantieni*	LD-3	KC209558		
*Tonkinomysdaovantieni*	HL-17	KC209559		
*Tonkinomysdaovantieni*	HL-22	KC209560		
*Tonkinomysdaovantieni*	HL-24	KC209561		
*Tonkinomysdaovantieni*	HL-25	KC209562		
*Tonkinomysdaovantieni*	HL-26	KC209563		
*Tonkinomysdaovantieni*	KIZ 201703366	MG865973		
*Tonkinomysdaovantieni*	KIZ 201703007	MG865972		
*Tonkinomysdaovantieni*	KIZ 201703005	MG865971		
*Tonkinomysdaovantieni*	KIZ 201611187	MG865970		
*Tonkinomysdaovantieni*	DPL 1662	MG189667	MG189683	
Outgroups				
*Saxatilomyspaulinae*		JQ755867		
*Saxatilomyspaulinae*		JQ755868		
*Leopoldamysneilli*		JX573331		
*Leopoldamysneilli*		JX573334		
*Chiromyscuschiropus*		KF154039		
*Chiromyscuschiropus*		KF154040		
*Niviventerconinga*		EF053025		
*Niviventerconinga*		EF053026		
*Niviventerconfucianus*		KY068809		
*Niviventerconfucianus*		KY068810		

**Table 3.** Estimates of evolutionary divergence between *Tonkinomysdaovantieni* geographical populations.

		Cao Bang	Terra typica	Yunnan	*Saxatilomys*	*Chiromyscus*	*Niviventer*	*Leopoldamys*
*Tonkinomys* populations	Cao Bang		0.00241	0.00523	0.02069	0.02478	0.02688	0.02658
	Terra typica	0.00892		0.00504	0.02061	0.02458	0.02713	0.02543
	Yunnan	0.02503	0.02285		0.02127	0.02778	0.02719	0.02503
relative genera	*Saxatilomys*	0.12955	0.12877	0.13681		0.02980	0.03107	0.02648
	*Chiromyscus*	0.17353	0.17322	0.19189	0.17863		0.02164	0.02501
	*Niviventer*	0.19768	0.20010	0.20051	0.19967	0.16416		0.02638
	*Leopoldamys*	0.18867	0.18221	0.18131	0.16434	0.16749	0.19548	

**Table 4.** Cranial and dental measurements for *Tonkinomysdaovantieni* populations. **Abbreviations**: (as indicated in Material and Methods).

Measurements	Holotype (Musser et al. 2006)	Terra typica group mean (Musser et al. 2006 n = 7-13)	SD	Yunnan group mean (Cheng et al. 2018n = 4)	SD	Terra typica group mean (our data for Huu Lien, n = 10)	SD	Cao Bang mean (this paper n = 2)	SD
**LHB (mm)**	213	204.6	10.34	183.25	15.13	192.2	17.27	180.5	18.40
**LT (mm)**	177	169.0	7.0	171.25	14.93	166.6	9.56	166.0	13.66
**LHF (mm)**	40	39.0	1.1	36.75	1.26	35.4	1.14	37.0	1.33
**LE (mm)**	30	30.0	0.8	30.25	1.71	30.2	0.84	28.5	0.84
**BW (g)**	160	171.0	22.8	124.25	43.04	187.48	38.55	137.95	40.6
**ONL (mm)**	51.2	50.0	1.6	48.35	2.53	48.54	2.35	44.46	2.177
**ZB (mm)**	21.6	21.3	0.2	20.58	1.25	20.87	0.71	18.775	1.34
**IB (mm)**	6.7	6.9	0.2	6.75	0.31	6.91	0.25	7.085	0.33
**LR (mm)**	17.7	17.1	0.6	16.93	1.18	17.53	1.27	15.695	1.03
**BR (mm)**	7.7	7.8	0.4	7.25	0.51	7.675	0.55	6.38	0.17
**BBC (mm)**	17.7	17.7	0.2	17.40	0.85	17.457	0.38	17,50	1.29
**HBC (mm)**	11.7	11.8	0.2	11.75	0.57	11.8	0.44	12.83	0.49
**BZP (mm)**	4.9	4.8	0.1	4.35	0.52	4.625	0.32	4.075	0.46
**LD (mm)**	13.3	13.6	0.5	12.93	1.10	12.545	0.88	11.50	1.02
**LIF (mm)**	9.1	9.5	0.5	8.08	0.67	9.095	0.41	7.675	1.17
**BIF (mm)**	3.4	3.5	0.2	3.13	0.24	3.395	0.18	2.99	0.03
**LBP (mm)**	10.7	10.5	0.3	10.65	0.52	10.255	0.62	10.37	0.06
**BBP (mm)**	4.6	4.6	0.1	4.20	0.55	4.495	0.36	4.39	0.06
**PPL (mm)**	16.2	15.7	0.6	14.63	1.02	14.83	1.08	12.95	0.72
**BMF (mm)**	3.9	3.8	0.2	3.8	0.29	3.73	0.18	3.465	0.15
**LB (mm)**	6.2	6.3	0.1	6.13	0.30	5.717	0.29	6.085	0.05
**CLM^1-3^ (mm)**	8.3	8.3	0.2	8.18	0.19	8.175	0.28	7.905	0.32
**BM (mm)**	2.1	2.1	0.1	2.03	0.10	1.9	0.08	1.80	0.07
